# Fœtal Hydrocephalus

**Published:** 1865-02

**Authors:** C. A. White

**Affiliations:** Iowa City, Iowa


### FŒTAL HYDROCEPHALUS.

By C. A. WHITE, M. D.

On the 16th of Dec., 1864, I was called to see Mrs. W. in labor, the messenger saying that "the body was born, but the head could not be, and the child was dead." I went immediately, taking my forceps with me. Arrived at 4½, P. M., and found the patient, a strong, healthy looking woman of 25 years, in her first labor, which she believed a month too soon, and in the condition as stated by the messenger. A midwife was in attendance, who stated that "labor commenced at 1, P.

M., and at 3 the waters broke, with the feet presenting; three pains following closely upon each other brought forth the body; the child was alive, and kicked many times." The pains, she said, then ceased entirely and the child soon died.

The patient was comparatively comfortable, the pulse good and no symptoms of exhaustion. Upon palpation of the abdomen it was evident that there was more in the womb than an ordinary sized head and the placenta. By examination per vaginam, I was able to reach the occiput, and by considerable effort got my index finger in the child's mouth, the face being at the left sacro-iliac synchondrosis, but could not move the head in the least. I informed the friends of the condition of affair, and said that I had little hope of delivering her without an operation. They were very averse to this, and as the woman's strength was good, and the pelvis capacious, I determined to give ergot.

Preparing a decoction of three drachms of the grains, of good quality, I gave it all to her in four fluid drachm doses, every fifteen or twenty minutes, without producing a single uterine pain. This was as far as I thought proper to carry its use, although the child was dead and not, of course, in any danger from violent contractions of the uterus, should they occur, and the uterus itself being partially contracted, in little or no danger of laceration, as I believed, from the same cause. After allowing sufficient time to elapse after the exhibition of the ergot, and finding the womb closely contracted over its contents, I decided to use the forceps. I had no difficulty in introducing them, but when introduced they would not lock. This confirmed my previous belief that the head was of abnornal shape and size. The forceps were of the pattern used by Prof. DeLaskie Miller of Rush Med. College.

By further palpation of the abdomen I could distinctly feel the disconnected bones of the cranium jutting upon one another, and believed that I could detect fluctuation when laying my left hand on the abdomen, and thrusting the index finger of my right, against the occipital bone.

It was now 1 o'clock at night, and twelve hours since labor commenced, the patient's strength was failing, and it was too far to send for my perforator: so taking the scissors from her work-basket, I thrust them through the occipital bone of the child, widened the wound with my finger, and drew off about two pints of limpid fluid, by estimate. The cranium collapsed like an empty bag, and immediately came away, followed at once by the placenta. The womb readily contracted to the usual size after ordinary delivery, without hemorrhage and without a single uterine pain having occurred after the shoulders were born.

No accident had befallen the mother during gestation, her health had been good during the whole period, and she has recovered as well from her confinement as is usual in favorable cases. Both the parents are, to all appearances, strong and healthy.

There was no separate tumor containing the fluid, but it was all contained within the expanded cranium, which was more than twice the normal size. The child was apparently well developed in other respects, and would have weighed seven or eight pounds if the cranium had contained nothing unusual, but I had no opportuuity of making a careful examination ot it.

Iowa City, Iowa, December 28th, 1864.

